# Enhancing Australian Mortality Data to Meet Future Health Information Demands

**DOI:** 10.3390/ijerph19010603

**Published:** 2022-01-05

**Authors:** James Eynstone-Hinkins, Lauren Moran

**Affiliations:** Australian Bureau of Statistics, Canberra 2616, Australia; james.eynstone-hinkins@abs.gov.au

**Keywords:** mortality data, cause of death, coronial investigation

## Abstract

The Australian mortality data are a foundational health dataset which supports research, policy and planning. The COVID-19 pandemic necessitated the need for more timely mortality data that could assist in monitoring direct mortality from the virus as well as indirect mortality due to social and economic societal change. This paper discusses the evolution of mortality data in Australia during the pandemic and looks at emerging opportunities associated with electronic infrastructure such as electronic Medical Certificates of Cause of Death (eMCCDs), ICD-11 and automated coding tools that will form the foundations of a more responsive and comprehensive future mortality dataset.

## 1. Introduction

The national mortality dataset (NMD) in Australia is produced annually by the Australian Bureau of Statistics (ABS). Data are sourced from the civil registration and vital statistics system, and provide a foundation for both population and heath research, policy and planning. As life expectancy has increased over the past century, the NMD has charted key changes in population health which have contributed to these gains. Key changes include large reductions in infant mortality rates [1], decreases in coronary artery disease mortality since 1968 [2], the aging of the population and the subsequent emergence of dementia as a leading cause of death [3].

The NMD has evolved and improved significantly over its long history. Many improvements are related to enhancements to the death registration process, with more thorough death investigation processes and recording of key demographic details leading to improvements in source data. Advancements in medical science and technology have led to improved diagnosis of diseases and cause of death certification. As knowledge about diseases and death has improved, the classification that underpins mortality data—the International Classification of Diseases (ICD)—has also been revised, leading to incremental changes in mortality datasets over time.

The NMD is part of a rich tapestry of health information data in Australia and has been more commonly used for epidemiological research and annual tracking of patterns of death. Health surveillance systems have produced rapid data for certain causes such as influenza, providing policy makers with timely information that can direct intervention and prevention activities when required. Health surveillance systems have also played the primary role in monitoring mortality due to COVID-19 during the pandemic.

As the COVID-19 pandemic has progressed there has been growing recognition of the importance of Civil Registration and Vital Statistics (CRVS) systems in filling key data gaps, as governments seek to understand its full impact on population health. Measures of excess mortality (the numbers of deaths that occur during a defined period compared to the number of expected deaths during that same period) help track both direct COVID-19 mortality and indirect mortality, for instance deaths which might relate to changes in access to health care [4]. In mid-2020 the ABS began releasing provisional mortality reports. These reports focused on all deaths that were doctor certified, and provided early indications of changes in patterns of mortality, including excess deaths, for key causes.

The demand for rapid mortality information continues to be driven by subsequent waves of COVID-19 and recognition that the longer-term effects of the pandemic need to be identified and addressed as early as possible. This is driving innovation in the way data is collected, analysed and used. This paper focuses on recent initiatives aimed at enhancing the NMD, and emerging opportunities associated with new electronic infrastructure that could shape the next generation of both health and mortality data.

## 2. Current Mortality Dataset Foundations

State and territory Registries of Births, Deaths and Marriages (RBDMs) are legislatively responsible for death registration in Australia. There are minor differences in form design and variables collected across jurisdictions, but the death registration process is generally consistent across the country. Death registrations require both a death registration statement (DRS) completed by an informant with the funeral director, and a medical certificate cause of death (MCCD) completed by a doctor. The format of the MCCD is based on the standard recommended by the World Health Organization. RBDMs lodge complete death registrations with the ABS for the compilation of the NMD.

Approximately 12% of deaths in Australia are referred to a coroner. While a death referred to a coroner must still follow the death registration process governed by the jurisdictional RBDMs, information pertaining to the cause of death is stored in the National Coronial Information System (NCIS). The NCIS is a medico-legal online database that holds information pertaining to coroner-referred deaths including police, toxicology, pathology and coronial reports [5]. This information is accessed by the ABS for coding of causes of death.

The DRS is used to inform the demographic component of mortality data with the cause of death coming from the MCCD or the NCIS. Cause of death certification is of high quality, with Australia found to have the lowest proportion of ‘unusable’ causes of death in a recent study focused on six high resource countries [6].

Australian cause of death information is coded using the 10th revision of the International Classification of Diseases (ICD-10). The automated coding system, Iris, is used assist with ICD-10 coding. Iris assigns ICD-10 codes to all terms on death certificates, then applies coding rules to select the underlying cause of death (the disease or condition that initiated the train of morbid events leading to death). Manual coding is required for most coroner referred deaths and doctor certified records unable to be processed by Iris.

Enhancements to the NMD are ongoing and target both demographic and cause of death variables. Enhancements to the demographic component are prioritised to support government priorities. The quality of identification of Aboriginal and Torres Strait Islander people on death registration documents is a high priority and has been a focus for both the ABS and RBDMs. More recently, place of death information was highlighted as a priority, with a framework subsequently developed to classify and capture this information [7].

Cause of death coding and output is also subject to frequent improvements. Since 1997, coded information on all causes listed on the death certificate (multiple cause data) has been retained. Multiple cause data provides important insights into the complex nature of death which has increasing relevance with an ageing population and people living with multiple co-morbidities which may collectively cause many pathways to death. This dataset is progressively being used to greater effect as methods for multiple cause analysis are strengthened, providing insights into relationships between diseases and their relative contributions to mortality.

Coronial investigative reports on the NCIS provide a rich source of information on circumstances and causes of coroner referred deaths. Risk factors for coroner referred deaths which are not a diagnosable disease but may have a detrimental impact on health are now coded as part of the NMD using ICD frameworks. Common risk factors relating to suicide deaths include previous self-harm attempts, disruption of family by separation or divorce, problems relating to economic or legal circumstances, unemployment and homelessness. Risk factors also vary with age, highlighting the importance of chronic health conditions and limitations to activity as key factors among older age groups [8].

### Rapid Reporting during the COVID-19 Pandemic

The COVID-19 pandemic created a demand for accurate and timely information on causes of death. The health surveillance system in Australia can provide rapid updates on COVID-19 mortality, but data only includes numbers of deaths and key demographics. It was recognized early in the pandemic that CRVS-based data would be able to provide important additional insights. Multiple cause data could provide insights into common comorbidities and consequences of the virus, while monitoring patterns of deaths across all causes could give insights into indirect effects of the pandemic or undiagnosed COVID-19 deaths.

There were challenges that needed to be addressed to enable rapid provision of CRVS-based mortality data during the pandemic. ICD coding challenges included the application of new rules for coding COVID-19 related deaths (rules were released by WHO in April 2020 and applied to all deaths where COVID-19 was mentioned on death certificates) and fast-tracking coding of all other deaths. Another key challenge was enabling meaningful comparison of rapid data against historical data. Investigations into issues of timeliness and completeness of data were conducted and guided key decisions on data releases.

In Australia, the time between when a death occurs and data lodgement is affected by the legislative requirement for burial or cremation prior to registration. While most doctor-certified deaths are registered within one month, it can be longer if the period between death and burial or cremation is prolonged. This period can be longer for coroner-referred deaths depending on circumstances and requirements of the coronial investigation.

System limitations necessitated scope changes for the production of the monthly NMD. These monthly outputs report on doctor-certified deaths only. The time between death and registration is shorter for doctor-certified deaths so more complete data is able to be published more rapidly. Data is also published by date of death to accurately measure mortality temporal to the pandemic. This differs to normal vital statistics reporting which is usually based on numbers of death registrations received in a specified period. Rapid data must be representative of the period of interest to enable meaningful comparison. Data in the monthly report is also considered to be provisional. This allows for deaths which are registered at a later time to be added once received [9].

Monthly provisional datasets use crude measures to highlight potential changes in patterns of mortality. A five-year average of raw counts of deaths is used as a proxy to measure expected deaths, with minimum and maximum numbers of deaths over that period provided to indicate a possible range. This method is applied to deaths from all causes and to specified causes of death. Age-standardised death rates with corresponding confidence intervals are also published to enable measurement of change over time.

Official calculations of excess mortality applied a robust regression to produce an expected number of deaths for 2020 [10]. Prediction intervals of 95% were also calculated. Only deaths which exceeded the upper bound of the prediction interval were considered to be excess. While excess mortality was recorded in some weeks, this was not sustained and, overall, during 2020, Australia did not record excess mortality. In the winter months, lower than expected mortality was recorded with the reduction reaching statistical significance. Measures in place to prevent the spread of COVID-19 likely led to a reduction in deaths from other causes, especially respiratory diseases. Similar patterns of mortality during the pandemic have been reported in New Zealand, Denmark and Norway [11].

COVID-19 has raised awareness of the importance of civil registration based mortality data in many countries, also leading to enhanced cooperation between agencies and accelerating the digitisation of registration services [12]. In Australia, it has driven a need to re-think some aspects of mortality data collection and reporting, highlighting new opportunities to further enhance the timeliness and relevance future datasets.

## 3. New Electronic Foundations for Mortality Data Systems

The COVID-19 pandemic has reinforced the importance of CRVS-based mortality data for both epidemiological study and for monitoring emerging public health concerns. Delivering on COVID-19 related data needs has helped identify barriers to rapid data production across the CRVS system, also highlighting where changes could result in significant improvements. In Australia, the delay between when deaths occur and when they are registered, time required to quality assure data and the complexity of cause of death coding, all contribute to delays in data availability.

The electronic transformation of the CRVS system provides an opportunity to streamline and further automate production of the NMD. In particular, the implementation of electronic Medical Certificates of Cause of Death (eMCCDs), the adoption of ICD-11 and the development and implementation of next generation auto-coding systems hold great potential to overcome existing system limitations.

### 3.1. Electronic Medical Certificates of Cause of Death

A key component of the electronic transformation of the CRVS system is the development of eMCCDs. These electronic forms have been developed by many countries and offer key advantages to paper forms. The instant digital capture of information supplied by a medical practitioner will reduce the time between when a death occurs and when information on that death can be made available. Electronic data capture also removes the need for transcription, improving timeliness and accuracy by eliminating transcription errors.

Early versions of eMCCDs in Australia follow similar formats to paper forms, but opportunities exist to enhance these products into the future. Electronic forms could improve the quality of certification by flagging sequencing errors or requesting additional information from the certifier. These forms could also link to the ICD foundations, potentially reducing error in data capture and enabling some degree of automated coding to occur during data collection.

### 3.2. ICD-11

The 11th revision of the ICD was adopted by the World Health Assembly in 2019 and is now available for implementation. ICD-11 was designed as an electronic classification. All entities including diseases, disorders, injuries and symptoms are stored in the ICD-11 foundation with each defined in a standard way using a structured content model. All entities also have their own URI, with a web-service API enabling direct links to other electronic infrastructure and health information systems [13]. These new capabilities go beyond the usual advancements in medical and scientific knowledge associated with an ICD revision and will make the classification a more integrated part of future health information infrastructure.

ICD-11 also enables capture of additional information about diseases and conditions using extension codes. In the mortality use case, extension codes may capture additional information on non-proprietary names of drugs for drug related deaths, or risk factors for external cause deaths. The possibilities associated with extension codes are extensive and may only be limited by the information available when coding and compiling data. Concepts such as post-coordination and clustering of codes have been proposed to provide structure to more complex ICD-11 datasets, although methods for structuring and using groups of codes will need further consideration.

### 3.3. Automated Coding Solutions

Auto-coding systems are critical for processing the large number of deaths that occur in Australia, allowing codes to be assigned to individual entities and automated rules to be applied for the selection of underlying causes of death. Australia uses the Iris mortality auto-coding system, with this product enabling auto-coding of around 65% of doctor certified deaths each year. A project is now underway to develop an ICD-11 version of Iris. This project seeks to realise the benefits of the extended vocabulary and concepts of ICD-11, to integrate with ICD-11 tools, to interface with healthcare systems and eMCCDs and use advanced techniques such as machine leaning to detect certification errors and increase auto-coding rates [14].

The new electronic components of the CRVS system hold the key to transforming information from death certificates into usable epidemiological data in a way that is rapid, automated, reliable and accurate, and consistent across institutions and countries [14], and will ensure mortality data can meet future information demands including those highlighted by COVID-19.

## 4. Conclusions

CRVS-based mortality datasets, including Australia’s NMD, are important epidemiological datasets that have guided health policy and planning for many years. The COVID-19 pandemic has highlighted the importance of rapid reporting of CRVS-based mortality data to complement data collected through surveillance systems and provide insights into the broader impacts of the pandemic beyond deaths directly from the virus.

Work undertaken in Australia to provide rapid data during the pandemic highlighted systemwide limitations that narrowed the scope of reported data. In particular, reporting needed to be sufficiently lagged to enable meaningful interpretation of changes in mortality, and only doctor-certified deaths could be included in reports. This limited the types of policy questions that could be addressed through rapid reporting to those concerning deaths from natural causes.

In Australia, electronic foundations already exist within the CRVS system with additional electronic components being developed and implemented by jurisdictional RBDMs. The electronic transformation of the CRVS system is expanding opportunities, with eMCCDs, ICD-11 and next-generation auto-coding tools likely to streamline future data collection, processing and reporting.

While COVID-19 has provided a catalyst for innovation and set clear new requirements for CRVS-based mortality datasets, the potential uses of rapid mortality data extend well beyond the pandemic. Rapid data will be able to provide early indications of changes in patterns of mortality relating to any number of events that could impact population health, including natural disasters, changes in natural cause patterns of mortality or infectious-disease-related epidemics.

## Data Availability

Not applicable.
